# Prevalence and predictors of antenatal depressive symptoms among Chinese women in their third trimester: a cross-sectional survey

**DOI:** 10.1186/s12888-015-0452-7

**Published:** 2015-04-02

**Authors:** Yingchun Zeng, Ying Cui, Jie Li

**Affiliations:** Department of Obstetrics, The Third Affiliated Hospital of Guangzhou Medical University, Guangzhou, China; Department of Psychiatry, The Third Affiliated Hospital of Guangzhou Medical University, Guangzhou, China; Guangzhou Brain Hospital, Guangzhou Medical University, 36# Mingxin Road, Liwan, Guangzhou China

**Keywords:** Pregnancy, Antenatal depression, Social support, Coping, Chinese women

## Abstract

**Background:**

Depression during pregnancy can be detrimental to both maternal and fetal health outcomes. A cross-sectional study was undertaken, with the goal of determining the prevalence and predicting factors associated with antenatal depressive symptoms during late pregnancy among Chinese women.

**Methods:**

Participants were recruited during bookings for antenatal care at a maternal and child health hospital’s outpatient care clinics. Measurements included the Chinese version of Self-rating Depression Scale, Eysenck Personality Questionnaire, Social Support Rating Scale, and Simplified Coping Strategies Questionnaire.

**Results:**

A total of 292 women participated in this study, with 28.5% prevalence of depressive symptoms. Significant protective predictors were: a younger age (OR = 0.85; 95% Confidence Interval-CI 0.76-0.95), good partner relationship (OR = 0.40; 95% CI 0.17-0.93), preparedness for delivery (OR = 0.36; 95% CI 0.20-0.63), active coping (OR = 0.92; 95% CI 0.89-0.96), and social support (OR = 0.92; 95% CI 0.88-0.97). In contrast, significant risk factors were: a history of miscarriage (OR = 1.86; 95% CI 1.30-2.66), irregular menstrual history (OR = 2.98; 95% CI 1.64-5.40), and financial worries (OR = 2.33; 95% CI 1.27-4.30). Psychosocial risk factors include psychoticism and neuroticism personality traits (OR = 1.06; 95% CI 1.02-1.10 and OR = 1.07; 95% CI 1.04-1.10, respectively), and pregnancy pressures (OR = 1.04; 95% CI 1.02-1.07).

**Conclusion:**

Depressive symptoms are common in third trimester antenatal clinic attendees. Interventions for early recognition of depression should target older women with a history of miscarriage and financial worries. Intervention strategies could be by providing more social support and promoting active coping strategies. Findings support a recommendation that antenatal services consider integrating screening for depression in routine antenatal care.

## Background

Antenatal depression has been identified as a serious health problem, but is a neglected component of care for women in the third trimester of pregnancy [1,2]. While postnatal depression is now well recognised [3], antenatal depression has not been studied extensively [4]. Antenatal depression is a depressive episode that begins in pregnancy, and is often a predictor of postnatal depression [5]. Research in the U.S. reported that 33% of postnatal depression begins in pregnancy [6]. In addition, some evidence indicates there could be a higher prevalence of depressive symptoms during pregnancy than in the postnatal period [7], as diagnosis of antenatal depression is difficult because the physiological signs of pregnancy overlap with the symptoms of antenatal depression [5]; and healthcare providers mainly focus on the physical health aspects of pregnancy [8].

The prevalence rate of antenatal depression has been reported at up to 42.7% [9]. Symptoms of antenatal depression include sadness during most of the day, hopelessness, lack of interest and fatigue, trouble sleeping and eating, along with extreme irritability and an inability to feel happiness or joy [10]. There is evidence that antenatal depression can have adverse effects on the mother, her child, and the family as a whole [2,11]. First, adverse effects for mothers include noncompliance with prenatal care; reduced sleep; clinical implications such as increased uterine irritability, pre-eclampsia, postpartum bleeding, preterm delivery and even suicide [1,10]. Second, negative outcomes associated with their babies include low birth weight, smaller head circumference, lower Apgar scores, and unhealthy behaviours; while among children they include smoking and alcohol consumption [1,12]. Children of depressed mothers have a high risk of emotional and behavioural problems and cognitive development delay [13]. Third, antenatal depression also has negative impacts on families. The most significant consequences of antenatal depression may negatively influence social and personal adjustment, marital relationships, and mother-infant interactions [14].

Risk factors for depression during pregnancy are largely similar to those for depression at other times, although treatment considerations for antenatal depression differ [2,10]. Research has indicated that some of the most common risk factors for antenatal depression include social support, life satisfaction and perceived stress [1]. A review of 57 studies indicated that some of the most important risk factors for antenatal depression are life stress, a history of depression, lack of social support, unintended pregnancy, Medicaid insurance, domestic violence, lower income, lower education, smoking, and single status [11,15]. Other studies have identified previous pregnancy loss, pregnancy problems, unplanned pregnancy and fear of childbirth as risk factors for antenatal depression [16]. There appears to be a paucity of research examining risk factors for antenatal depression among Chinese women.

Given the high prevalence of antenatal depression and its serious consequences, efforts should be made to identify risk factors to assist in prevention, identification and treatment [16]. Research literature on antenatal depression is limited, especially in developing countries such as China. There is a lack of research regarding antenatal depression and its predicting factors. Knowledge of risk factors for antenatal depression in clinical settings can alert healthcare providers to the possibility of a depressive disorder [14]. This study, therefore, aims to identify the prevalence of third trimester antenatal depressive symptoms and its predictors among Chinese women.

## Methods

A cross-sectional survey was used to identify the prevalence of antenatal depressive symptoms among Chinese pregnant women in their third trimester and its predicting factors.

### Participants

Participants were primiparous mothers recruited from antenatal clinics in a maternal and child hospital in South China. The antenatal phase comprised 292 participants consecutively recruited over three months. Eligible criteria for participants were adult women (18 years old or older), in their third trimester of pregnancy. Multiparous women and women in the first and second trimester were excluded.

### Measures

#### General information sheet

The general information sheet consisted of 20 items, including information on demographic characteristics such as age, geographic location, years of education, monthly income, medical payment types and a number of questions on obstetric information such as gestational age, history of miscarriage or pregnancy termination, maternal and fetus health status, desired fetus gender, preparedness for delivery, and any financial concerns related to medical costs.

#### Self-rating Depression Scale (SDS)

The SDS was originally developed by Zung [17], and is one of the most widely used self-rating measures for clinicians to identify depressive symptoms in adults [18]. This scale is a 20-item Likert-style (4-point) rating scale for depression, with a theoretical score range extending from 20 to 80. The original total score plus 1.25 will be the standardised score (<50 as normal; 50–59 as mild depressive cases; 60–69 as moderate depressive cases; and ≥70 as severe depressive cases) [19]. The Chinese version of SDS is widely used among Chinese women during pregnancy [20,21]. Gao et al. [21] also proposed taking the standardised score of 50 as an appropriate cut-off in screening depressive symptoms for Chinese women during pregnancy.

#### Eysenck Personality Questionnaire (EPQ)

Personality traits of the women were evaluated by the EPQ, which consists of three scales to measure Extraversion, Neuroticism, and Psychoticism [22]. Each item is responded to using a dichotomous (yes/no) response format. High scores in Extraversion reflect sociability, assertiveness, and the tendency to experience positive emotions (23 items). High scores in Neuroticism reflect moodiness, worry, and the tendency to experience negative emotions (24 items). High scores in Psychoticism reflect impulsiveness, tough-mindedness, and emotional detachment (32 items). In this study, the internal consistency coefficients were generally satisfactory for all factors: 0.79 for Neuroticism, 0.81 for Extraversion, and 0.71 for Psychoticism. The test-retest reliability of EPQ from a study in the UK was 0.86 for Extraversion, 0.78 for Neuroticism, and 0.79 for Psychoticism [23]. Validation was based on confirmatory factor analysis, which revealed a sufficient fit of an oblique factor solution testing the three original EPQ dimensions [24].

#### Pregnancy Pressure Scale (PPS)

Pregnancy pressure levels were measured using the PPS. The PPS consists of 30 items and four sub-domains: PPS1-Pressure related to the transition to parenthood; PPS2-Pressure related to concern for maternal and infant safety; PPS3-Pressure related to changes in body image and limitations in physical activity; PPS4-Pressure related to other sources of pregnancy [25]. Responses to each question ranged from 0 (never) to 4 (very often). All items in each sub-domain were added to a sub-total score. Higher scores indicate a high pressure level. The PPS has demonstrated an acceptable reliability among Chinese women [25]. The internal consistency by Cronbach’s alpha of PPS in this study was 0.92.

#### Simplified Coping Style Questionnaire (SCSQ)

The Chinese version of SCSQ was used to assess participants’ coping strategies. The SCSQ is a 20-item scale with two dimensions: “active coping” (12 items) and “passive coping” (8 items) [26]. Active coping emphasises positive coping characteristics, such as ‘handling the distressing emotions caused by the problem’. Passive coping emphasises the characteristics of negative coping, such as ‘escaping troubles by drinking and smoking’ [26]. A higher score for each dimension indicates frequent use of the coping style. The internal consistency of the SCSQ by Cronbach’s alpha was 0.81 for the active coping subscale and 0.76 for the passive coping subscale.

#### Social Support Rating Scale (SSRS)

The SSRS was originally developed in China by Xiao [27]. This scale was adopted to evaluate participants’ social support. The SSRS is comprised of 10 items and three domains: objective support (3 items), subjective support (4 items) and support use degree/the use of social support (3 items). Higher scores indicate better social support from family, friends and significant others. The SSRS was widely used in assessing social support for Chinese women [28]. In this study, the internal consistency of SSRS was 0.89 by Cronbach’s alpha.

### Data collection

Prior to commencement of data collection, ethics approval was granted from the Ethical Committee of the Maternal and Child Health Hospital. Informed consent was gained from participants prior to questionnaire completion. Data were collected from January to March 2013. Midwives with trained research skills recruited women consecutively during their routine antenatal visit.

### Data analysis

Data analysis was performed using IBM SPSS (version 20.0). Descriptive statistics were used to summarise participants’ demographic and clinical characteristics, and the prevalence of antenatal depressive symptoms by frequency and percentages, and to describe the mean scores of measured outcomes by means and standard deviation. Independent t-tests were used to find differences between non-depressive and depressive women in terms of measured outcomes. Binary logistic regression analysis was conducted to identify predictors of antenatal depressive symptoms. *P*-values less than 0.05 were regarded as statistically significant.

## Results

### Subject characteristics

Out of 350 pregnant women approached, a total of 292 women agreed to participate in this study, with a response rate of 83.4%. The mean age was 24.18, with women ranging in age from 19 to 33. All participants were in their third trimester. More than half had had one or more miscarriages. Most women were located in rural areas (n = 238, 81.5%), with monthly family incomes ranging from 1,000 to 3,000 RMB (n = 214, 73.2%). More than half of the women had Medicaid insurance (n = 179, 61.3%). However, 59 women (20.2%) still reported financial worries about their medical costs. Other detailed demographic and medical characteristics are listed in Table 1.

**Table 1:** **Demographic and obstetric characteristics of participants (N = 292)**

**Characteristics**	**Mean (SD)**	**n (%)**
**Age** (Years)	24.18 (2.59)	
**Week of pregnancy**	34.71 (2.45)	
**Number of miscarriages**	0.74 (0.73)	123 (42.1% as 0)
**Home location**		
Urban		54 (18.5)
Rural		238 (81.5)
**Education levels**		
Primary school or below		142 (48.6)
High school		108 (37.0)
College or above		42 (14.4)
**Monthly income** (Yuan)		
<1000		18 (6.2)
1000-2000		121 (41.4)
2001-3000		93 (31.8)
>3000		60 (20.5)
**Medical expenses**		
Medicaid insurance		179 (61.3)
Self-payment		113 (38.7)
**Partner relationship**		
Good		270 (92.5)
General		22 (7.5)
**Maternal health status**		
Good		270 (92.5)
General		22 (7.5)
**Menstruation history**		
Regular		229 (78.4)
Irregular		63 (21.6)
**Antenatal health check**		
Regular		280 (95.9)
Irregular		12 (4.1)
**Fetus health worries**		
Yes		258 (88.4)
No		34 (11.6)
**Desired fetus sex worries**		
Yes		258 (88.4)
No		34 (11.6)
**Preparedness of delivery**		
Yes		218 (74.7)
No		74 (25.3)
**Financial worries**		
Yes		59 (20.2)
No		233 (79.8)

### Prevalence of antenatal depression and mean scores of predictors

As seen in Table 2, the prevalence of antenatal depressive symptoms was 28.5%, and the degree of depressive symptoms ranged from mild to severe. Means of depression scores by SDS was 43.47 (10.83), Median of SDS was 41.25, and Skew and Kurtosis were .709 and .002, respectively. Means of predictors are listed in Table 3. Most of the women were classified as stable in personality. The most common source of pressure perceived by pregnant women concerned maternal and infant safety, followed by various pressure sources related to their transition to parenthood. Women were more likely to use a positive coping tendency. In terms of social support by SSRS, participants perceived better objective support than subjective support.

**Table 2:** **Classification of possible depression cases (N = 292)**

**Variables**	**n (%)**
**Self-rating depression scale** (25–100)	
Normal cases (<50)	209 (71.5)
Mild cases (50–59)	53 (18.2)
Moderate cases (60–69)	23 (7.9)
Severe cases (≥70)	7 (2.4)

**Table 3:** **Mean scores of predicting factors (N = 292)**

**Instruments**	**Mean (SD)**
**Eysenck Personality Questionnaire (EPQ)**	
Psychoticism or Stability	43.88 (7.15)
Extraversion or Introversion	51.90 (8.62)
Neuroticism or Stability	47.02 (9.84)
**Pregnancy Pressure Scale (PPS)**	
PPS1-Pressure related to transition to parenthood	3.11 (3.38)
PPS2-Pressure related to concerning on maternal and infant safety	7.62 (4.77)
PPS3-Pressure related to changes of body image and limitation of physical activity	2.75 (2.88)
PPS4-Pressure related to other sources of pregnancy	1.70 (1.71)
**Simplified Coping Style Questionnaire (SCSQ)**	
Active coping strategies	1.55 (0.77)
Passive coping strategies	0.99 (0.48)
**Social Support Rating Scale (SSRS)**	
Subjective support	8.62 (2.44)
Objective support	24.36 (4.06)
Use of social support	7.74 (1.85)

### Differences in measured health outcomes between normal and depressive women

Independent t-tests were used to examine differences in measured health outcomes between women with no depressive symptoms and with depressive symptoms (Table 4). In terms of personality, women with depressive symptoms were more likely to suffer from psychoticism (p = 0.001) and neuroticism (p < 0.001). Women with depressive symptoms had higher levels of pregnancy pressures, related to the transition to parenthood, than women without depressive symptoms (p < 0.001). The coping tendencies of women with depressive symptoms were more likely to be passive (p = 0.016). Women with depressive symptoms reported lower levels of objective support (p < 0.001) and degree of support use (p = 0.039).

**Table 4:** **Differences of measured outcomes between women with and without depressive symptoms (N = 292)**

**Instruments**	**Mean (SD)**		**In-dependent** ***t*** **-test**	
	**No depressive symptoms (n = 209)**	**With depressive symptoms (n = 83)**	**t**	***P***
**EPQ**				
Psychoticism	43.23 (7.15)	46.79 (6.42)	−3.33	.001
Extraversion	51.38 (8.85)	52.21 (7.57)	−0.28	.773
Neuroticism	45.95 (9.14)	51.89 (11.41)	−4.08	<.001
**PPS**				
PPS1	2.76 (3.12)	4.70 (4.04)	−3.86	<.001
PPS2	7.63 (4.85)	7.58 (4.42)	0.06	.949
PPS3	2.62 (2.92)	3.32 (2.68)	−1.60	.110
PPS4	1.70 (1.71)	1.70 (1.69)	0.00	.998
**SCSQ**				
Active coping	1.63 (0.79)	1.16 (0.55)	4.08	<.001
Passive coping	0.85 (0.46)	1.02 (0.48)	−2.41	.016
**SSRS**				
Subjective support	25.30 (13.56)	23.92 (4.45)	0.72	.468
Objective support	8.91 (2.29)	7.32 (2.68)	4.42	<.001
Use of social support	7.85 (1.80)	7.26 (2.00)	2.07	.039

*Abbreviations*: *EPQ* eysenck personality questionnaire, *PPS* pregnancy pressure scale, *PPS1*-pressure related to transition to parenthood, *PPS2*-pressure related to concerning on maternal and infant safety, *PPS3*-pressure related to changes of body image and limitation of physical activity, *PPS4*-pressure related to other sources of pregnancy, *SCSQ* simplified coping style questionnaire, *SSRS* social support rating scale.

### Predictors of antenatal depressive symptoms among Chinese women during pregnancy

Binary logistic regression analyses were used to identify relevant predictors for antenatal depressive symptoms among Chinese women in their third trimester. The adjusted logistic regression odds ratios (OR) and 95% confidence intervals (CI) are reported in Table 5. Significant protective predictors were: a younger age (OR = 0.85; 95% CI 0.76-0.95), good partner relationship (OR = 0.40; 95% CI 0.17-0.93), preparedness for delivery (OR = 0.36; 95% CI 0.20-0.63), active coping (OR = 0.92; 95% CI 0.89-0.96), and social support (OR = 0.92; 95% CI 0.88-0.97). In contrast, significant risk factors were: a history of miscarriage (OR = 1.86; 95% CI 1.30-2.66), irregular menstrual history (OR = 2.98; 95% CI 1.64-5.40), and financial worries (OR = 2.33; 95% CI 1.27-4.30). Psychosocial risk factors include psychoticism and neuroticism personality traits (OR = 1.06; 95% CI 1.02-1.10 and OR = 1.07; 95% CI 1.04-1.10, respectively), and pregnancy pressures (OR = 1.04; 95% CI 1.02-1.07).

**Table 5:** **Predictors of antenatal depressive symptoms by adjusted logistic regression**

**Variables**	**B**	**SE**	**OR**	**95% CI**	***p***
**Demographic and clinical factors**					
Age	-.157	.056	0.85	0.76-0.95	.005
Week of pregnancy	-.017	.055	0.98	0.88-1.09	.751
Number of miscarriages	.623	.183	1.86	1.30-2.66	.001
Home location (urban, rural)	-.290	.334	0.74	0.38-1.44	.385
Education levels (primary school, ≥high school)	-.061	.059	0.94	0.83-1.05	.304
Monthly income (<3000, ≥3000)	-.315	.160	0.73	0.53-0.99	.049
Medical expenses (insurance, self-payment)	.255	.190	1.29	0.88-1.87	.180
Partner relationship (general, good)	-.916	.435	0.40	0.17-0.93	.035
Maternal health status (good, general)	-.568	.403	0.56	0.25-1.24	.158
Menstruation history (regular, irregular)	1.093	.303	2.98	1.64-5.40	<.001
Antenatal health check (regular, irregular)	.530	.787	1.69	0.36-7.93	.501
Fetus health worries (yes, no)	1.007	.551	2.73	0.93-8.05	.067
Desired fetus sex worries (yes, no)	-.046	.431	0.95	0.41-2.22	.915
Preparedness of delivery (no, yes)	−1.021	.292	0.36	0.20-0.63	<.001
Financial worries (no, yes)	.849	.311	2.33	1.27-4.30	.006
**Psychosocial factors**					
EPQ-Psychoticism	.065	.019	1.06	1.02-1.10	.001
EPQ-Extraversion	-.003	.016	0.99	0.96-1.02	.832
EPQ-Neuroticism	.073	.015	1.07	1.04-1.10	<.001
PPS total score	.047	.013	1.04	1.02-1.07	<.001
SCSQ-Active coping	-.076	.020	0.92	0.89-0.96	<.001
SCSQ-Passive coping	-.009	.035	0.99	0.92-1.06	.806
SSRS total score	-.075	.022	0.92	0.88-0.97	.001

*Abbreviations*: *EPQ*, eysenck personality questionnaire, *PPS* pregnancy pressure scale, *SCSQ* simplified coping style questionnaire, *SSRS* social support rating scale.

## Discussion

This study provides a cross-sectional snapshot of the prevalence and relevant predictors of antenatal depressive symptoms among Chinese women in their third trimester. The prevalence of antenatal depressive symptoms found in the current study was 28.5%. A meta-analysis of 21 studies concluded the mean prevalence of depression across the antenatal period was 10.7% [29]. Hence, the prevalence of antenatal depressive symptoms in this study was higher than in those studies in both developed and developing countries. It is possible that poor identification and measurement of depression during pregnancy directly contributes to some depressive women being misclassified as postpartum onset [2].

For demographic risk factors, being in a younger age group was a statistically protective factor of antenatal depressive symptoms. This is consistent with previous research by Koleva, and colleagues [11], who found that an older age was a significant predictor of antenatal depression. Obviously, pregnancy at an older age, especially pregnancy higher than 35 years old, is usually categorized as high risk pregnancy, so older women are more likely to be vulnerable and feel depressed. In relation to the predictor factor of miscarriage history, Koleva, and colleagues [11], found that the number of previous pregnancies was significantly associated with antenatal depression. While previous research suggests the strongest risk factor for antenatal depressive symptoms was a history of depression [30], the present study lacked information on any past psychiatric episode, and whether there was a family history of depression.

In terms of the psychosocial predictors of antenatal depressive symptoms, both personality traits of psychoticism and neuroticism were significant risk factors (OR = 1.06 and 1.07, respectively). Consistent with other research [16,31], active coping and total social support were significant protective factors for antenatal depressive symptoms. Hence, active coping strategies and social support would be possible mediators between demographic and clinical risk factors and antenatal depressive symptoms among Chinese pregnant women. Findings in this study can inform healthcare providers to adjust antenatal care accordingly with a more psychological aspect for care, such as training pregnant women in more active coping strategies to obtain more social support.

Fostering social support could be carried out through community development strategies, including community participation. For example, community peer support group interventions could be useful strategies in helping women obtain adequate support as they enter motherhood. While the development of perinatal mental health services in China is still in its infancy, integrated models of perinatal mental health service delivery - including identification and prevention, which are in use in developed countries - could be used to guide Chinese perinatal mental health service development [2]. Furthermore, integrating perinatal mental health into routine maternity care should emphasise a primary health care approach. In cases of severe depression, antenatal care providers should refer patients to psychiatrists, in order to obtain specialist care. While some women will require referral to a psychiatrist, greater emphasis should still be placed on setting up community support systems that adequately support women.

Several study limitations should be taken into account. Study participants were restricted to women in their third trimester of pregnancy, therefore study findings cannot be generalised to the first and second trimesters. In addition, this study adopted a cross-sectional study design precluding causal conclusions. Longitudinal study designs are needed to monitor these depressive women and establish the causality of predictors for antenatal depressive symptoms. Another study limitation was the under-representation of women who were primiparous, married, and mainly coming from rural areas. Future research replicating this study with more multiparous urban pregnant women would be beneficial for the representativeness of the study population.

Despite these limitations, study findings have important implications. For practice, antenatal care providers should routinely screen all pregnant women for depressive symptoms, as a U.S. study has found that 33% of postnatal depression begins during pregnancy [6]. Additionally, antenatal care providers should refer women at high risk of depression to psychiatric services whenever necessary [1]. Targeted interventions for antenatal depression may reduce both symptom severity of depression and assist in the amelioration of pregnancy pressures. Implementation and empirical validation of targeted antenatal interventions are proposed for future research [16]. For education, relevant curricula for maternity care courses should include routine screening tools for antenatal depression, and incorporate evidence-based health care lectures on depression during pregnancy [1].

## Conclusions

Depressive symptoms were relatively high in hospital antenatal attendees in a suburban city in South China. While active coping strategies and social support were significant protecting factors of depressive symptoms, significant risk factors include older age, a history of irregular menstruation and/or miscarriage, financial worries, and pregnancy pressure. Personality traits of psychoticism or neuroticism were also significant risk factors of depressive symptoms. Pregnancy brings major challenges for any women’s physical, social and psychological health. Due to the prevalence of depression during pregnancy and its negative impacts on both mother and infant, mental health should be made an integral part of routine obstetric care in China. Possible strategies could include close collaboration between psychiatry services and routine preventive obstetric services, and routinely conducting antenatal screening for depression and other psychiatric illness.

## References

[1] Abujilban SK, Abuidhail J, Al-Modallal H, Hamaideh S, Mosemli O (2014). Predictors of antenatal depression among Jordanian pregnant women in their third trimester. Health Care Women Int.

[2] Howard LM, Molyneaux E, Dennis CL, Rochat T, Stein A, Milgrom J (2014). Non-psychotic mental disorders in the perinatal period. Lancet.

[3] Misri S (2007). Suffering in silence: the burden of perinatal depression. Can J Psychiatry.

[4] van Hyssteen L (2008). Need for wider recognition of antenatal depression. Ther Today.

[5] Harvey ST, Pun PK (2007). Analysis of positive Edinburgh depression scale referrals to a consultation liaison psychiatry service in a two-year period. Int J Ment Health Nurs.

[6] Wisner KL, Sit DK, McShea MC, Rizzo DM, Zoretich RA, Hughes CL (2013). Onset timing, thoughts of self-harm, and diagnoses in postpartum women with screen-positive depression findings. JAMA Psychiatry.

[7] Heron J, O’Connor TG, Evans J, Golding J, Glover V, ALSPAC Study Team (2004). The course of anxiety and depression through pregnancy and the postpartum in a community sample. J Affect Disord.

[8] Strass P, Billay E (2008). A public health nursing initiative to promote antenatal health. Canadian Nurse.

[9] Imran N, Haider II (2010). Screening of antenatal depression in Pakistan: risk factors and effects on obstetric and neonatal outcomes. Asia-Pacific Psychiatry.

[10] Humayun A, Haider II, Imran N, Iqbal H, Humayun N (2013). Antenatal depression and its predictors in Lahore, Pakistan. East Mediterr Health J.

[11] Koleva H, Stuart S, O’Hara MW, Bowman-Reif J (2011). Risk factors for depressive symptoms during pregnancy. Archives Women’s Mental Health.

[12] Lusskin SI, Pundiak TM, Habib SM (2007). Perinatal depression: Hiding in plain sight. Can J Psychiatry.

[13] Bonari L, Pinto N, Ahn E, Einarson A, Steiner M, Koren G (2004). Perinatal risks of untreated depression during pregnancy. Can J Psychiatry.

[14] Kaaya SF, Mbwambo JK, Kilonzo GP, Van Den Borne H, Leshabari MT, Fawzi MC (2010). Socio-economic and partner relationship factors associated with antenatal depressive morbidity among pregnant women in Dares Salaam, Tanzania. Tanzan J Health Res.

[15] Lancaster C, Gold KJ, Flynn HA, Yoo H, Marcus SM, Davis MM (2010). Risk factors for depressive symptoms during pregnancy: a systematic review. Am J Obstetrics Gynecol.

[16] Leigh B, Milgrom J (2008). Risk factors for antenatal depression, postnatal depression and parenting stress. BMC Psychiatry.

[17] Zung WW (1965). A self-rating depression scale. Arch Gen Psychiatry.

[18] Ruiz-Grosso P, Loret de Mola C, Vega-Dienstmaier JM, Arevalo JM, Chavez K, Vilela A (2012). Validation of the Spanish center for epidemiological studies depression and Zung self-rating depression scales: a comparative validation study. PLoS One.

[19] Lee HC, Lee HC, Chiu HF, Wing YK, Leung CM, Kwong PK (1994). The Zung Self-rating depression scale: screening for depression among the Hong Kong Chinese elderly. J Geriatr Psychiatry Neurol.

[20] Hu Y (2011). Analysis on the anxiety and depression status and influencing factors of pre-delivery pregnant women. Master Thesis.

[21] Gao Y, Wang W, Huang D, Liu H (2014). Analysis of anxiety and depression status and influencing factors among parturient pre-delivery pregnant women. Anhui Med J.

[22] Eysenck HJ, Eysenck SBG (1975). Manual of the Eysenck Personality Questionnaire.

[23] Smillie LD, Bhairo Y, Gray J, Gunasinghe C, Elkin A, McGuffin P (2009). Personality and the bipolar spectrum: normative and classification data for the Eysenck personality questionnaire-revised. Compr Psychiatry.

[24] Rothen S, Vandeleur CL, Lustenberger Y, Jeanprêtre N, Ayer E, Fornerod D (2009). Personality traits in children of parents with unipolar and bipolar mood disorders. J Affect Disord.

[25] Zhang ZJ (2001). Manuals of behavioral medicine measure.

[26] Xie Y (1998). Reliability and validity of the simplified coping style questionnaire. Chinese J Clin Psych.

[27] Xiao SY (1994). The social support rating scale. Psychological health rating scale manual.

[28] Yi J, Zhong B, Yao S (2014). Health-related quality of life and influencing factors among rural left-behind wives in Liuyang, China. BMC Women’s Health.

[29] Dennis CL, Dowswell T (2013). Interventions (other than pharmacological, psychosocial or psychological) for treating antenatal depression. Cochrane Database of Systematic Review.

[30] Rich-Edwards JW, Kleinman K, Abrams A, Harlow BL, McLaughlin TJ, Joffe H (2006). Socio-demographic predictors of antenatal and postpartum depressive symptoms among women in a medical group practice. J Epidemiol Community Health.

[31] Lau Y, Yin L, Wang Y (2010). Antenatal depressive symptomatology, family conflict and social support among Chengdu Chinese women. Matern Child Health J.

